# Laparoscopy in Emergency: Why Not? Advantages of Laparoscopy in Major Emergency: A Review

**DOI:** 10.3390/life11090917

**Published:** 2021-09-03

**Authors:** Giuseppe Ietto, Francesco Amico, Giuseppe Pettinato, Valentina Iori, Giulio Carcano

**Affiliations:** 1 Emergency and Transplant Surgery Department, ASST-Settelaghi and University of Insubria, 21100 Varese, Italy; valentina.iori@gmail.com (V.I.); giulio.carcano@uninsubria.it (G.C.); 2 Trauma Service, Department of Surgery, University of Newcastle, Newcastle 2308, Australia; Francesco.amico@hotmail.com; 3 Department of Medicine, Beth Israel Deaconess Medical Center, Harvard Medical School, Boston, MA 02115, USA; gpettina@bidmc.harvard.edu

**Keywords:** laparoscopy, emergency surgery, trauma, immune system, DAMPs, multiple organ dysfunction, ARDS

## Abstract

A laparoscopic approach is suggested with the highest grade of recommendation for acute cholecystitis, perforated gastroduodenal ulcers, acute appendicitis, gynaecological disorders, and non-specific abdominal pain (NSAP). To date, the main qualities of laparoscopy for these acute surgical scenarios are clearly stated: quicker surgery, faster recovery and shorter hospital stay. For the remaining surgical emergencies, as well as for abdominal trauma, the role of laparoscopy is still a matter of debate. Patients might benefit from a laparoscopic approach only if performed by experienced teams and surgeons which guarantee a high standard of care. More precisely, laparoscopy can limit damage to the tissue and could be effective for the reduction of the overall amount of cell debris, which is a result of the intensity with which the immune system reacts to the injury and the following symptomatology. In fact, these fragments act as damage-associated molecular patterns (DAMPs). DAMPs, as well as pathogen associated molecular patterns (PAMPs), are recognised by both surface and intracellular receptors of the immune cells and activate the cascade which, in critically ill surgical patients, is responsible for a deranged response. This may result in the development of progressive and multiple organ dysfunctions, manifesting with acute respiratory distress syndrome (ARDS), coagulopathy, liver dysfunction and renal failure. In conclusion, none of the emergency surgical scenarios preclude laparoscopy, provided that the surgical tactic could ensure sufficient cleaning of the abdomen in addition to resolving the initial tissue damage caused by the “trauma”.

## 1. Introduction

Why should laparoscopy be preferred in emergency?

The history of laparoscopy in emergency and urgent surgical scenarios, both traumatic and pathological, began immediately after the implementation of the technique itself.

Less than 20 years after the first experiment of laparoscopy on living dogs by Kelling in 1901 [1], the description concerning the use of laparoscopy for diagnosing traumatic hemoperitoneum appeared in the literature [2,3]. In 1924, laparoscopy was suggested as a technique for diagnosing internal bleeding from trauma [3] and in 1925 for detecting blood after traumatic viscera rupture [2].

In the 1940s, laparoscopy was considered to be contraindicated for acute abdominal illnesses and stab wounds, gunshot wounds, and acute perforations of viscera, for fear of spreading infection [4], although by this time it had already been accepted in its early stages as enabling a precise diagnosis [5].

The modern concept of diagnostic laparoscopy for trauma began in the 1960s, when Heselson proclaimed its safety, efficacy and economic benefits, and demonstrated decreased hospitalisation, avoiding unnecessary laparotomies [6,7,8,9].

The advantages of laparoscopy in several emergency situations are clearly stated, undoubtedly whenever the surgical procedure is standardised. On the other hand, the benefits are lesser or unclear when only a basic plan of the procedure is definable and the surgical technique strictly depends on the intra-abdominal findings. 

Our aim is to review all of the evidence concerning the choice of laparoscopy in emergency settings and to clarify the pathomechanism underlying the choice.

The available evidence clearly demonstrates the superiority of a laparoscopic approach in many emergency situations, with the grade of recommendation outlined in Table 1. 

In these scenarios, laparoscopy guarantees the main advantage of having a shorter hospital stay that is associated with a quicker surgery, which is that patients experience a faster and uneventful recovery.

Earlier enteral feeding is related to such a fast recovery. Adequate nutritional support contributes to maintaining the homeostasis and, consequently, optimises the immune response, minimising the risk of post-surgical infection [37].

In terms of contraindication, these do not differ from those clearly defined for elective surgery in the case of an abdominal emergency. 

Laparoscopy should cautiously be considered whenever there is suspected difficulty in accessing the abdomen, such as in cases of organomegaly, adherence syndrome, or bowel distension.

However, the additional systemic and local effects of laparoscopy must be mentioned because these may represent limitations in some emergency scenarios.

In fact, the creation of the pneumoperitoneum is a key part of the procedure. The main consequences are due to increased intra-abdominal pressure, which causes respiratory, cardiovascular, and neurological alterations. Cardiovascular effects depend on intra-abdominal pressure and carbon dioxide uptake into the systemic circulation. At intra-abdominal pressures greater than 15 mmHg, the inferior vena cava is compressed, causing a reduction in venous return and resulting in a decrease in cardiac output and blood pressure. Increased intra-abdominal pressure elevates the diaphragm, and diminishing lung volume may cause basal atelectasis. The effects of the pneumoperitoneum and patient positioning may also raise intracranial pressure, reducing cerebral perfusion [38,39]. Thus, the general complications of laparoscopy are cardiac compromise, cerebral complications, pneumonia, and urinary tract infection [40]. Absolute and relative contraindications to laparoscopy in the approach to abdominal emergencies are the same as for elective procedures; in general, stability of hemodynamic and respiratory parameters are required to perform laparoscopic procedures. Major peritonitis should not be considered a contraindication to laparoscopy. Traumatised or critically ill patients may be eligible if sustained stability of hemodynamic and respiratory parameters is achieved after resuscitation and/or intensive medical treatment [35,41].

On the other hand, the pneumoperitoneum could precipitate pre-existing abnormalities in cardiac output or gas exchange, as well as intracranial pressure, liver dysfunction or coagulopathy.

## 2. Acute Cholecystitis

Acute cholecystitis is an acute inflammatory disease of the gallbladder, most commonly due to gallstones. Cholecystectomy is the accepted treatment for acute calculous cholecystitis and is considered to be the standard of care for gallstone disease for the majority of patients [10]. The laparoscopic approach was initially considered to be contraindicated for acute cholecystitis. Increases in surgical experience led to the laparoscopic approach becoming the preferred procedure, even in complicated settings. Critical patient conditions, such as septic shock or anaesthesiology contraindications, are reasons to avoid laparoscopic cholecystectomy. Regarding the timing of surgery, the safety and efficacy of early and delayed laparoscopic cholecystectomy for acute cholecystitis are comparable. Early laparoscopic cholecystectomy (ELC) may be more technically demanding and time-consuming and may be associated with a higher rate of wound infections but is associated with a reduced hospital stay and risk of readmissions. In the latest guidelines from the World Society of Emergency Surgery it is stated that ELC is superior to either intermediate laparoscopic cholecystectomy, performed between seven days and six weeks of hospital admission, or delayed laparoscopic cholecystectomy which performed between six weeks and three months of the initial hospital admission. ELC should be the standard of care whenever possible, even for patients at high risk for surgery [11]. A recent meta-analysis of randomised clinical trials by Borzellino et al., failed to confirm the hypothesis that immediate cholecystectomy, performed within 24 h of admission, may reduce post-operative complications unless surgery could be performed within 72 h of the onset of symptoms [12]. A recent systematic review, comparing open versus laparoscopic cholecystectomy, revealed that the laparoscopic approach is associated with a lower complication rate and with a shorter hospitalisation period. There were no differences for the same-admission cholecystectomy in terms of morbidity, operative time, intraoperative blood loss and the rate of bile leakage. Laparoscopy is also associated with a decrease in mortality rate, postoperative hospital stay, wound infection and pneumonia. The operative time is shorter for the laparoscopic approach [12,13]. Tang et al., in a systematic review, identified risk factors for conversion. These include male gender, old age, severe obesity, cirrhosis, previous upper abdominal surgery, severe acute and chronic cholecystitis, and emergency laparoscopic cholecystectomy [14]. The absence of the critical view of safety (CVS) is the most important factor for conversion to open surgery. Mandatory indicators for conversion include a complete buried gallbladder, an impacted stone and the inability to retract the gallbladder [42]. In cases of severe inflammation, the identification and the dissection of the Calot’s triangle could be difficult. In such situations a laparoscopic fundus first anterograde approach is used to avoid bile duct injury [43]. An intraoperative cholangiography could be performed to better clarify the anatomy or in case of a suspicion of a common bile duct stone. Furthermore, a recent meta-analysis suggests that using near-infrared fluorescent cholangiography with indocyanine green intraoperatively decreases the rate of some complications, as compared with bile duct injury and conversion-to-open-surgery rates relative to cholecystectomy under white light alone [44]. An alternative solution that could be used in particularly adverse conditions is the execution of a subtotal cholecystectomy. Subtotal cholecystectomies are divided into “fenestrating” and “reconstituting” types. Subtotal reconstituting cholecystectomy is a procedure in which the surgeon creates a remnant gallbladder, closing its lower part. This will lead to a major risk of recurrence of symptomatic cholelithiasis but will minimise the risk of fistula. Subtotal fenestrating cholecystectomy requires the internal closure of the cystic duct. This procedure has a higher incidence of postoperative biliary fistula but does not seem to be associated with recurrent cholecystolithiasis [45,46]. Laparoscopic subtotal cholecystectomy represents a good solution that leads to a decrease in conversion rates, without increasing the incidence of postoperative complications [47].

## 3. Gastroduodenal Ulcer

Gastroduodenal perforation is an acute abdominal disease most commonly caused by peptic ulcers with an incidence of 2–10% [15]. The treatment of peptic ulcers has widely changed in recent years, and the surgical approach is now performed to treat the complications. The results of some clinical trials suggest that laparoscopic surgery could be a better strategy than open surgery in the correction of perforated peptic ulcers but the evidence is not strongly for or against this intervention [16]. Laparoscopy is preferred in patients with no risk factors, whereas in higher risk classes it leads to a major rate of mortality and conversion [17]. The LAMA randomised multi-centre trial by Bertleff et al. shows a conversion rate of about 8% [18]. The most common causes for conversion are due to the size of the perforation, friable margins of the lesion, the severity of peritonitis, the state of shock at the time of hospitalisation and the delay in the diagnosis >24 h. The operating time for laparoscopy is higher, despite the experience gained over the last decade, which has led to a progressive reduction in operating times. The most frequent complication is suture leakage. The systematic review conducted by Lunevicius and Morkevicius revealed no statistically significant difference in the incidence of reintervention between the laparoscopic approach and the open technique, although the incidence was doubled after laparoscopy (5.3% vs. 2.1%) [19]. A recent meta-analysis from Cirocchi et al. compared laparoscopic to open surgery for patients with perforated peptic ulcers [20]. They reported a significant advantage of laparoscopic repair in terms of postoperative pain in the first 24 h after surgery and regarding the incidence of wound infections. No significant differences between laparoscopic and open surgery were found for overall postoperative mortality, leak of the suture repair, intra-abdominal abscesses and reoperation rate. This suggests that it is reasonable to utilise a laparoscopic approach for stable patients and in the presence of appropriate surgical skills [21]. The laparoscopic approach is an important diagnostic tool, allowing for defining the ulcer in its location, size and, in some cases helping to establish the aetiology of the ulcer. An exploratory laparoscopy could be performed where there is suspicion of a perforated gastroduodenal ulcer, based especially on the clinical history and physical examination of the patient. An indication for surgical exploration is the evidence of sickle air at the base of the right hemidiaphragm on the direct radiograph of the abdomen, or at the CT scan of the abdomen with a diagnostic accuracy of 98% [48]. After the exploratory laparoscopy, if a perforated ulcer is found, the surgeon could perform a laparoscopic repair. According to the latest guidelines from the WSES, sutureless repair has not gained wide acceptance because of the high leakage rate compared to suture repair. In patients with a perforated peptic ulcer smaller than 2 cm, it is recommended that a primary repair is performed. The application of the omental patch is a matter of debate and it is not the current standard of care; in fact, it has been reported to have similar results compared to simple suturing and is also related to longer operative times [21]. Nevertheless, the omental patch could be used in the case of a large ulcer with friable margins [49]. In such cases, there is the suspicion of malignancy and it is suggested that performing a frozen pathologic examination and resection. The kind of resection and the subsequent reconstruction differs basically upon the site of the ulcer [50,51]. A key moment in the intervention is the cleansing of the abdominal cavity; the specific amount of saline fluids is not precisely defined and the positioning of drainage is not mandatory [52]. The laparoscopic approach is gaining importance in diagnosis and the treatment of complicated peptic ulcers, and one of the most relevant prognostic factors is the degree of experience achieved by surgical teams. However, further studies are needed to better define this aspect, with particular reference to laparoscopic learning curves. 

## 4. Acute Appendicitis

Acute appendicitis is the leading cause of emergency surgery. In developed countries, occurs in 5.7–50 patients per 100,000 inhabitants per year, especially at the ages of 10–30 years [22,23]. The first appendectomy was codified by McBurney [24] and the first laparoscopic assisted appendectomy was performed in 1977 by Hans de Kok [25]. Today the laparoscopic approach is the most effective surgical treatment for acute appendicitis [23]. Several systematic reviews of randomised control trials comparing laparoscopic with open appendectomy have reported that the laparoscopic approach is associated with longer operative times and higher operative costs, but it leads to less postoperative pain, lower rates of surgical site infection and shorter hospital stays [25,26]. In addition, the laparoscopic approach is the favourable treatment for obese, elderly and pregnant patients [27,28]. In cases of uncomplicated acute appendicitis, patients can be treated with antibiotics as a first approach, in selected patients and in cases of the exclusion of gangrenous acute appendicitis, abscesses and diffuse peritonitis [53]. The five-year follow-up results of the APPAC randomised trial supports the feasibility of non-operative management with antibiotics as an alternative to surgery [54]. The laparoscopic approach is a safe and efficient procedure, superior or comparable to open appendectomy in terms of several surgical outcomes for both uncomplicated and complicated appendicitis [55,56]. The laparoscopic appendectomy is feasible for complicated appendicitis, in which it could reduce the hospital stay and lower the risk of surgical site infection. It also appears to have significant benefits with lower morbidity compared to the open approach [57]. It is still unclear whether it is appropriate in the event of intra-abdominal abscess, due to low certainty of the evidence available [58].

## 5. Gynaecologic Emergencies

In cases requiring emergency surgery the laparoscopic approach for gynaecologic conditions has the advantage over laparotomy of shorter hospitalisation and a faster recovery. In addition, it permits a complete exploration of the abdominal cavity, allowing the treatment of different diseases through using the same access, reducing the risk of post-surgical adhesions and surgical site infections. The main gynaecologic conditions that may present to the general surgeon in an emergency scenario are pelvic inflammatory disease (PID), ovarian diseases, ectopic pregnancy and endometriosis. Different treatments are available for PID, although surgery is required especially if there is no improvement in clinical conditions or in the presence of a pelvic abscess. The laparoscopic approach is indicated to perform the lavage and drainage of the infected collections, adhesiolysis and debridement of necrotic tissue [59]. In the case of ectopic pregnancy, the surgical treatment depends on different factors related to the patient, together with the size of pregnancy and the condition of the uterine tubes. The desire for future pregnancy is also an important factor that must be considered. Some factors would contraindicate the laparoscopic approach, particularly in the case of pregnancy located in the broad ligament, corneal pregnancies larger than 1 cm or if rupture occurs [60,61]. Laparoscopy allows for a shorter hospital stay, lower operative time and an earlier return to normal activities. Adnexal torsion is an uncommon cause of pelvic pain and typically involves the ovaries or uterine tubes [62]. The common presentation is acute onset of pelvic pain, associated with nausea, vomiting, fever and an increase in the leukocytes count. Immediate surgery, favourable with laparoscopy, is required. The laparoscopic approach should be favoured as it allows both for conservative treatment, by the detorsion and adnexectomy if necessary. In cases of recurrent torsion stabilisation of the adnexa by suture at the pelvic side wall can be performed. The shortening of the utero-ovarian ligament by plication and suturing is also used [63,64]. Concerning endometriosis, surgery aims to remove ectopic tissues in order to restore the anatomy, but it is rare to perform emergency surgery for pelvic pain, unless it is secondary to an operation being performed for hemoperitoneum or for suspected appendicitis. The removal of endometriosis requires the careful manipulation of the organs involved, especially in the case of infiltration of the serosa of the bowel, above the urethra and bladder and above major blood vessels [65].

## 6. Nonspecific Abdominal Pain

Nonspecific abdominal pain is not a defined disease; it refers to an abdominal or pelvic pain of a duration of less than one week for which the diagnosis remains uncertain even after clinical examination and baseline investigations [33,34]. NSAP is a very common condition. Selective indication to laparoscopy after a short period of active observation reduces the need for surgery without significant clinical disadvantages in patients affected by NSAP [35]. Whether an early laparoscopy (EL) or active observation should be preferred is largely debated. Analysis of the available data shows that EL has several advantages over active observation in terms of cost-effectiveness and a shorter hospital stay with a specific diagnosis [66]. Open surgery has a limited role in the management of NSAP; it is associated with low diagnostic accuracy, high morbidity and costs and long hospital stay. An significant proportion of patients (3–20.7%) are discharged without a diagnosis, and up to 28.8% of patients complain of persisting NSAP [36]. 

When clinical examination and radiological findings can only suspect the disease responsible for the acute abdomen, laparoscopy allows for wide exploration of the abdomen to ascertain the diagnosis. In the international literature, accuracy is reported with a rate of 89–100% [35,67,68,69,70].

In such situations and whenever, despite a clear intra-abdominal picture, the surgical management cannot be standardised, but the tactic must be adjusted to the intraoperative findings, the therapeutic role of laparoscopy is a matter of debate (Table 2).

A laparoscopic approach might guarantee significant advantages only if performed by experienced teams and surgeons, guaranteeing a high standard of care.

The laparoscopic approach can also be advantageous in abdominal trauma in hemodynamically stable patients. In fact, it could allow for a quicker diagnosis and could also limit blood loss and reduce the risk of contamination. 

## 7. Abdominal Trauma

In recent years, indications for laparoscopy in abdominal trauma have widely increased, but the procedure is still reserved for hemodynamically stable patients. The laparoscopic approach is useful in treating bleeding from minor injuries of the liver or the spleen [116,117], mesentery and hollow viscus injuries [118], and for diaphragmatic repair of small and isolated injuries [119,120,121]. It is generally indicated in cases of penetrating thoracoabdominal and abdominal trauma, with documented or equivocal penetration of the anterior fascia, gunshot wounds with doubtful intraperitoneal trajectory and in cases of non-operative management with a progressive worsening of clinical, laboratory and imaging data. Laparoscopy is the treatment of choice in trauma patients with free blood fluid in the peritoneal cavity where the source of bleeding cannot be determined [122]. The use of the laparoscopic approach in blunt trauma and in the case of complex injuries [123] is limited and debated [124]. In a prospective randomised study of 43 patients with abdominal stab wounds, however, there was no difference between laparotomy and laparoscopy strategies in terms of hospital costs [125]. In a recent comparative observational prospective cohort study conducted on isolated blunt abdominal trauma, laparoscopy was found to be a good alternative to laparotomy, as it is considered to be a reliable and safe method in hemodynamically stable patients. It is associated with a shorter hospital stay and lower rate of morbidity and mortality [126]. Complications of laparoscopy related to trauma occur in up to 11% of patients [124,127,128]. Possible complications are: tension pneumothorax in patients with diaphragmatic injury [127,129]; gas embolism in patients with intra-abdominal venous injuries; and metabolic and hemodynamic changes, such as acidosis, cardiac suppression, atelectasis, subcutaneous emphysema and increased intracranial pressure.

## 8. Paediatric Emergencies

The laparoscopic approach has gained importance in treating paediatric patients, not only in terms of elective procedures, but also in emergencies scenarios. With an experienced surgical team, a minimally invasive approach could be used safely in children, with low rates of morbidity and mortality [130]. Laparoscopy is safe and effective in the management of complicated Meckel diverticulum in performing the diverticulectomy, minimising the incision, and for the evaluation of the degree of damage and the extension of gangrene [131]. In the case of failure of non-operative management, the laparoscopic reduction of intussusceptions is the treatment of choice in hemodynamically stable children [132]. Furthermore, laparoscopy should be the initial approach in the absence of volvulus in malrotations [133]. Minimally invasive surgery is becoming the elective approach in the treatment of enteric duplication cysts, although the treatment of asymptomatic patients and the timing of surgery in cases of antenatal diagnose remain controversial. Early excision in asymptomatic patients is associated with less morbidity and a shorter hospital stay, compared to symptomatic patients. There are significant post-operative morbidities after resection of complicated cysts, compared with its elective surgery [134].

## 9. Pathophysiological Considerations

To understand the reasons that guide the surgical tactic, pro or contra laparoscopy, it is fundamental to know the pathological mechanisms underlying the outcome of the patient. 

We support the idea of minimising post-surgical infection, not only with the use of antibiotic prophylaxis, but also through the optimisation of the immune response by maintaining the homeostasis with nutritional support (especially enteral, as the laparoscopy ensures a faster GI recovery allowing an earlier enteral feeding) and reducing surgical trauma (as is done with laparoscopy). These measures consequently reduce the stress response and immune suppression [37].

To this concern, ten years ago the European Association for Endoscopic Surgery (EAES) stated, as subsequently reported:

“Changes in systemic inflammatory and anti-inflammatory parameters (mainly cytokines) as well as in stress response parameters are less pronounced after laparoscopic surgery than conventional surgery (grade A).”

However, the EAES notes that it remains to be proven if this leads to clinically relevant effects.

In fact, the extremely broad range of symptomatology developed after trauma or any significant severe acute disease, seems to be correlated with the intensity through which the immune system reacts, this is also related with the severity of the initial tissue damage caused by the “trauma”.

In other words, any abdominal urgency, as well as traumatic injury events, almost instantaneously (or at least extremely quickly) causes a high level of cell death, with the consequential release of cell debris fragments. These events deploy the so-called damage-associated molecular patterns (DAMPs) derived from cellular necrosis, and the pathophysiological consequences follow the mechanistic insight depicted in the so-called “danger model”.

The “danger model”, described by Matzinger in 2002, is based on the idea that the immune system does not distinguish between self and non-self, but rather between events that cause damage and events that will not [135,136,137]. In other words, the immune system does not distinguish between self and non-self, but discriminates between dangerous and safe, by recognising pathogens or alarm signals from injured or stressed cells and tissues.

This model represents the last evolution of the scientific concept regarding the activation of the immune system, which takes its origin from the traditional “non-self model”, developed in 1959, and passes through the “infectious non-self model” after the rediscovered concept of co-stimulation by Jenkins and Schwartz in 1986 [136]. 

For both the “infectious non-self model” and the “danger model”, antigen-presenting cells (APCs) play an important role in the activation of both the innate immune system and the acquired immune response.

As proposed by Janeway in 1989 [138], APCs are quiescent until they are activated via pattern-recognition Receptors (PRRs) that recognise conserved pathogen-associated molecular patterns (PAMPs) on bacteria and fungi and damage-associated molecular patterns (DAMPs), such as mtDNA, histones and other cell fragment debris that act as an alarm, and initiate the immune response. PRRs are both on the surface of APCs and inside the cytoplasm and are called Toll-like receptors (TLRs) [139,140] and nucleotide-binding oligomerisation domain (NOD)-like receptors (NLRs) [141], respectively.

Concurrently both PAMPs and DAMPs bind to the nucleotide-binding oligomerisation domain (NOD), which together with leucine-rich repeat (LRR) and pyrin domains containing protein 3 (NLRP3) constitute the subunit of the inflammasome. The activation of the NOD domain causes the oligomerisations of NLRPs that is the first step towards the activation of the inflammasome. Once activated the NLRP3 inflammasome leads to caspase 1-dependent cleavage of pro IL-1β and pro IL-18 into their active forms, as well as to gasdermin D-mediated pyroptotic cell death [142,143].

The mediator of lung inflammation, fever and fibrosis is IL-1β. DAMPs spreading after pyroptosis amplifies the cascade and potentiates the inflammation.

Mt-DNA is one of the most investigated and best described DAMPs [144], being both a marker and initiator of sterile post-injury inflammation. It is released by cell necrosis and it can also be expelled through an active process by intact functioning mitochondria following a cell stress response to major trauma [145,146]. Mitochondria and their products can turn into an enemy within the body, as a “Trojan horse”.

The activation of APCs up-regulates costimulatory signals, processes the foreign antigens, and presents them to passing T cells. In addition, cytokines or pathogen-associated molecular patterns (PAMPs) leads to the transcriptional upregulation of the canonical and non-canonical inflammasome components [147].

Between the causes of triggering the inflammatory response and beside the danger molecules, the formation of neutrophil-associated extracellular traps (NETs) seems to play an emerging role.

Neutrophil function and NETs are critical components involved in human immune defence. This was first described by Brinkmann et al. as a cell death pathway, which is distinct from apoptosis and necrosis [148,149], whereby neutrophils enter tissues and organs following an injury to form a neutrophil extracellular traps (NETs) [150,151,152]. 

NETosis leads to the dissolution of the nuclear envelope and cytoplasmic granules, leading chromatin to mix with granular antimicrobial proteins via the NADPH oxidase and RAf- MEK-ERK pathway [153]. 

NETs consist of neutrophil DNA, granular proteins, and several cytoplasmic proteins with antimicrobial activity, including histones, neutrophil elastase, MPO, pentraxin (PTX), lactoferrin, cathepsin G, and bactericidal permeability increasing protein [148,149,154]. 

Although NETs play important roles in host defence by trapping pathogens, extensive formation of NETs with increased amounts of extracellular DNA may contribute to the perpetuation of inflammation and tissue damage [150,152,154,155,156,157,158,159,160,161]. Moreover, recent studies suggest that NETs could be related to thrombosis and can damage the endothelium of blood vessels [160,161,162]. NET constituents can activate platelets and promote an excessive coagulopathy and thrombosis, resulting in endothelial cell injury and organ damage [163].

Several studies have shown that for severe trauma or burn injury the expression of the leukocyte transcriptome will be altered during the first 28 days after injury. This “genomic storm” involves genes implied in the systemic inflammation, in the innate immunity response and the simultaneous suppression of those involving adaptive immunity, especially in terms of T cell function and antigen presentation. Much later, alteration of gene pathways occurs, which implicates B cell proliferation and immunoglobulin synthesis. Only at a later time will the pathway responsible for compensatory anti-inflammatory response, which is involved in suppression of adaptive immunity, be redefined. 

Interestingly, according to the severity of the injury, the magnitude and the duration of this physiological derangement differs and will become responsible for the development of multiple organ dysfunction syndrome (MODS), as the result of excessive pro-inflammatory response (systemic inflammatory response syndrome [SIRS]) followed temporally by compensatory anti-inflammatory response syndrome (CARS) and the suppression of adaptive immunity [164,165,166].

The patho-mechanisms are consistent with a genomic storm that is neither chaotic nor erratic, but rather highly coordinated and reproducible. This storm likely represents a common transcriptional response to severe stress in humans regardless of its origin, with far more similarities than differences [167].

In summary, in critically ill surgical patients a deranged response to the addicted infectious insult and to the first amount of PAMPs and DAMPs is a trigger for subsequent activation of downstream pathways. The results are the development of a progressive and multiple organ dysfunctions manifesting with ARDS, coagulopathy, liver dysfunction and renal failure. 

The predominant presentation depends on the underlying prevalent pathomechanism: thrombotic microangiopathies and coagulopathies and/or complement pathways’ hyper activation that may lead to disseminated microvascular thrombosis; pathologic immune activation and following hyper inflammation; immune paralysis with associated secondary bacterial or fungal infections and an inability to combat inflammation due to CD4+, CD8+ and dendritic cells deficiency. 

In the light of this overview and following the basic principles of any efficacious treatment after an abdominal acute emergency, there is not only the ability to resolve the injury itself, but also the capability to clear the damaged tissues which limits the release of DAMPs.

In addition, a longer surgical procedure might be justified to obtain this goal, enabling a faster recovery.

Taking into account all of these considerations, none of the emergency surgical scenarios preclude laparoscopy. 

In fact, the main advantage is its capability of limiting tissue damage, which, if associated with adequate cleaning of the abdomen, could ensure its superiority to an open approach [168,169,170].

Laparoscopy should not be recommended only when unable to control the whole abdominal cavity due to both the extent of the disease and technical limitations due to the surgical instruments or to the experience of the team.

## 10. Conclusions

In conclusion, laparoscopy must be considered a safe, extremely versatile and prompt surgical approach, through which we could achieve a quicker diagnosis, avoiding large preoperative studies, minimising morbidity and requiring shorter hospital stay.

To this end, laparoscopic training in elective scenarios should be recommended, not only for the surgeon but for the entire team, including scrub nurses and the other members of the staff in the operating theatre must become confident.

Routine practice, combined with adequate equipment, could guarantee a high standard of care for laparoscopy in almost all emergency scenarios.

## Figures and Tables

**Table 1:** Overview of latest evidence for laparoscopy in emergency.

Laparoscopy Must Be the Preferred Approach in the Following Urgent Surgical Scenarios:	
Acute cholecystitis	[10,11,12,13,14]
Gastroduodenal perforated ulcers	[15,16,17,18,19,20,21]
Acute appendicitis	[22,23,24,25,26,27,28]
Gynaecological disorder	[29,30,31,32]
Non-specific abdominal pain (NSAP)	[33,34,35,36]

**Table 2:** Overview of minor evidences sustaining laparoscopy in emergency.

Laparoscopy Can Be Attempted but Not Strictly Recommended in the Following Scenarios:	
Acute pancreatitis	[71,72,73,74,75,76,77,78,79,80]
Diverticulitis	[81,82,83,84,85]
Adhesive small bowel obstruction	[86,87,88,89,90,91,92,93,94,95]
Incarcerated hernia	[35,96,97,98,99,100]
Acute mesenteric ischemia	[101,102,103,104,105]
Intestinal bleeding	[106,107,108,109,110,111,112,113]
Surgical and endoscopic complications	[114,115]

## Data Availability

Not applicable.
